# Street trees provide an opportunity to mitigate urban heat and reduce risk of high heat exposure

**DOI:** 10.1038/s41598-024-51921-y

**Published:** 2024-02-13

**Authors:** Ailene K. Ettinger, Gregory N. Bratman, Michael Carey, Ryan Hebert, Olivia Hill, Hannah Kett, Phillip Levin, Maia Murphy-Williams, Lowell Wyse

**Affiliations:** 1 The Nature Conservancy of Washington, 74 Wall Street, Seattle, WA 98121 USA; 2 School of Environmental and Forest Sciences, University of Washington, Seattle, WA 98195 USA; 3 Department of Environmental and Occupational Health Sciences, University of Washington, Seattle, WA 98195 USA; 4 Urban Forest Program, City of Tacoma, Tacoma, WA USA; 5 School of Marine and Environmental Affairs, University of Washington, Seattle, WA 98195 USA; 6 Tacoma Tree Foundation, Tacoma, WA USA

**Keywords:** Urban ecology, Climate change, Public health

## Abstract

Climate change is exacerbating the need for urban greening and the associated environmental and human well-being benefits. Trees can help mitigate urban heat, but more detailed understanding of cooling effects of green infrastructure are needed to guide management decisions and deploy trees as effective and equitable climate adaptation infrastructure. We investigated how urban trees affect summer air temperature along sidewalks within a neighborhood of Tacoma, Washington, USA, and to what extent urban trees reduce risks of high summer temperatures (i.e., the levels regulated by state outdoor heat exposure rules intended to reduce heat-related illnesses). Air temperature varied by 2.57 °C, on average, across our study area, and the probability of daytime temperatures exceeding regulated high temperature thresholds was up to five times greater in locations with no canopy cover within 10 m compared to those with 100% cover. Air temperatures decreased linearly with increasing cover within 10 m, suggesting that every unit of added tree cover can help cool the air. Our findings highlight the value of trees in mitigating urban heat, especially given expected warming with climate change. Protecting existing urban trees and increasing tree cover (e.g., by planting street trees), are important actions to enhance climate change resilience of urban areas.

## Introduction

Urban trees are increasingly recognized as valuable infrastructure for improving ecological and social resilience of cities. Urban greening has been linked to mitigation of heat islands (i.e., temperature reduction), improved air and water quality, reduction of stormwater run-off, biodiversity benefits, and enhanced human health and well-being^1–8^. These nature-based benefits are likely to become even more critical as climate change progresses, extreme heat events increase in frequency, urban areas expand, and urban populations increase^9–11^. Recognizing the benefits that trees can provide, many cities have set goals for urban tree canopy cover, and increasing urban tree cover has been proposed as a climate adaptation strategy^12^.

In temperate regions, the cooling benefits of tree canopy are particularly important in the summer, when high temperatures pose a health risk to urban residents and workers. The frequency of extreme high temperatures is increasing in cities, with record-setting heat reported in many northern hemisphere cities in recent years^9,13^. Heat exposure, especially during extreme heat events, is a public and occupational health concern, as it can cause heat stroke, heat exhaustion, fainting, and other potentially fatal heat related illnesses^14–16^. For example, during June 26–28, 2021, high temperature records were broken in several cities in the Pacific Northwest region of the United States and southwestern Canada (Oregon, Washington, British Columbia), resulting in increases in emergency calls, hospital visits, and deaths from heat-related illnesses^16^. The frequency and severity of such extreme heat events is expected to dramatically increase in a world with 2 °C of global warming above pre-industrial temperatures (i.e., 0.8 °C warmer than today^16^).

Trees may be able to help mitigate risks of heat stress to humans by reducing urban temperatures. Trees mitigate the urban heat island effect primarily through shading and transpiration. By blocking incoming solar radiation, tree shading can strongly reduce temperatures (e.g., by 3.06 °C, on average, in cities across the contiguous United States^17^), with the effect size varying regionally, depending on tree morphological characteristics and other factors^18^. Cooling effects from transpiration on temperatures also depend on tree characteristics, and can vary seasonally, during extreme climatic events, and in different urban and geographical contexts^18^.

The cooling benefits of trees have been widely described but remain inequitably distributed across cities. Historically marginalized communities that have experienced disinvestment, such as those that were “redlined” in cities across the United States, generally have lower tree canopy cover and hotter temperatures^19–22^. Indeed, a recent analysis found that the average person of color lives in census tracts with higher surface urban heat island intensity (a proxy for additional heat exposure) than non-Hispanic whites in nearly all large, urbanized areas in the continental United States^23^. These inequities cause severe harm: heat mortality rates are often higher in low-income neighborhoods and neighborhoods of color^24,25^. Addressing these environmental justice issues through interventions in the built and natural environment is an urgent need^26^.

To deploy tree planting most effectively as a tool to address environmental injustices and health risks posed by increasing temperatures, there is a need to better understand and quantify effects of urban tree planting at local scales (i.e., the scales at which humans experience them). Many studies quantifying effects of trees on temperature occur at continental scales, often correlating remote-sensed metrics of tree canopy with land surface temperature or modelled air temperature, e.g.,^27^. Though helpful for identifying the potential benefits of different amounts of canopy cover and for understanding landscape-scale patterns, these broad-scale studies may not reflect local patterns in the environmental conditions that humans are experiencing (e.g., variation in air temperature at fine spatial scales not captured by these estimates, or air temperature versus remote-sensed measures of land surface temperature). These fine-scale data are necessary to inform tree planting and management decisions (e.g., presence of powerlines or compacted gravel may affect management type and frequency or planting opportunities), which typically occur at local scales (e.g., the municipality level).

**Figure 1:**
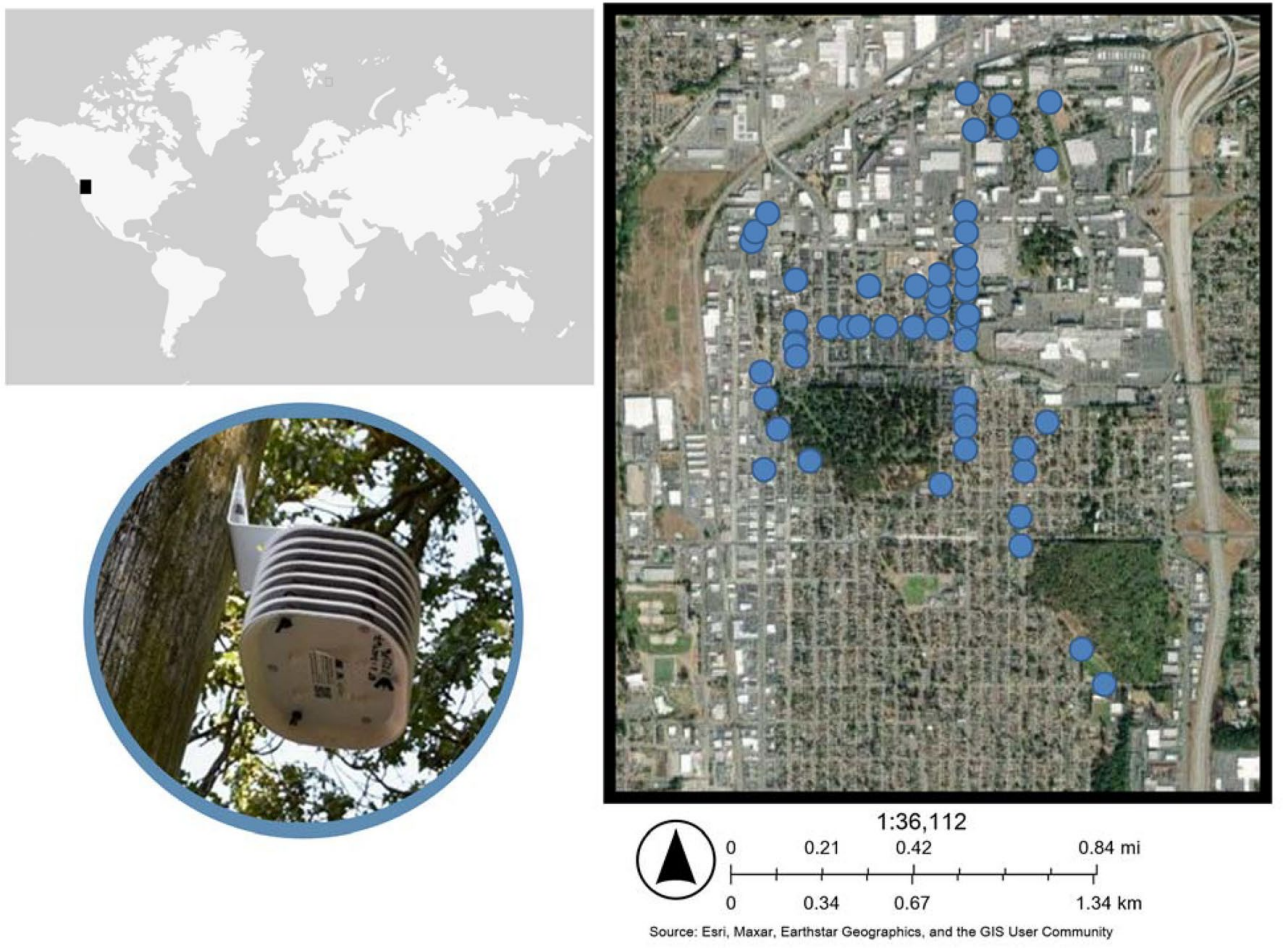
Our study area for this work, which is part of the Greening Research in Tacoma (GRIT) project, is located in South Tacoma, Washington, USA, shown by the black square on global map, where solar radiation shields (photographed) containing temperature loggers were installed on utility poles. Here we report on temperature data from loggers at 46 locations throughout the neighborhood (blue dots) during summer 2022.

Here we evaluate effects of urban trees on air temperatures in a neighborhood in Tacoma, Washington, USA, where we established an air temperature monitoring network (Fig. 1). We focus on temperature during June, July, and August as these are the hottest months in our study region. This neighborhood currently experiences lower than city-wide average tree canopy cover and higher summer temperatures^28^. Specifically, we asked:

1. How do trees affect summer air temperature in South Tacoma, and which metrics of tree abundance best explain variation in temperature?
2. To what extent do urban trees reduce risks of human exposure to dangerously high temperatures?
3. How do tree canopy metrics derived from remote-sensed data compare with field-measured metrics of tree canopy cover and other vegetation cover in their ability to explain variations in summer air temperature?

## Results

**Figure 2:**
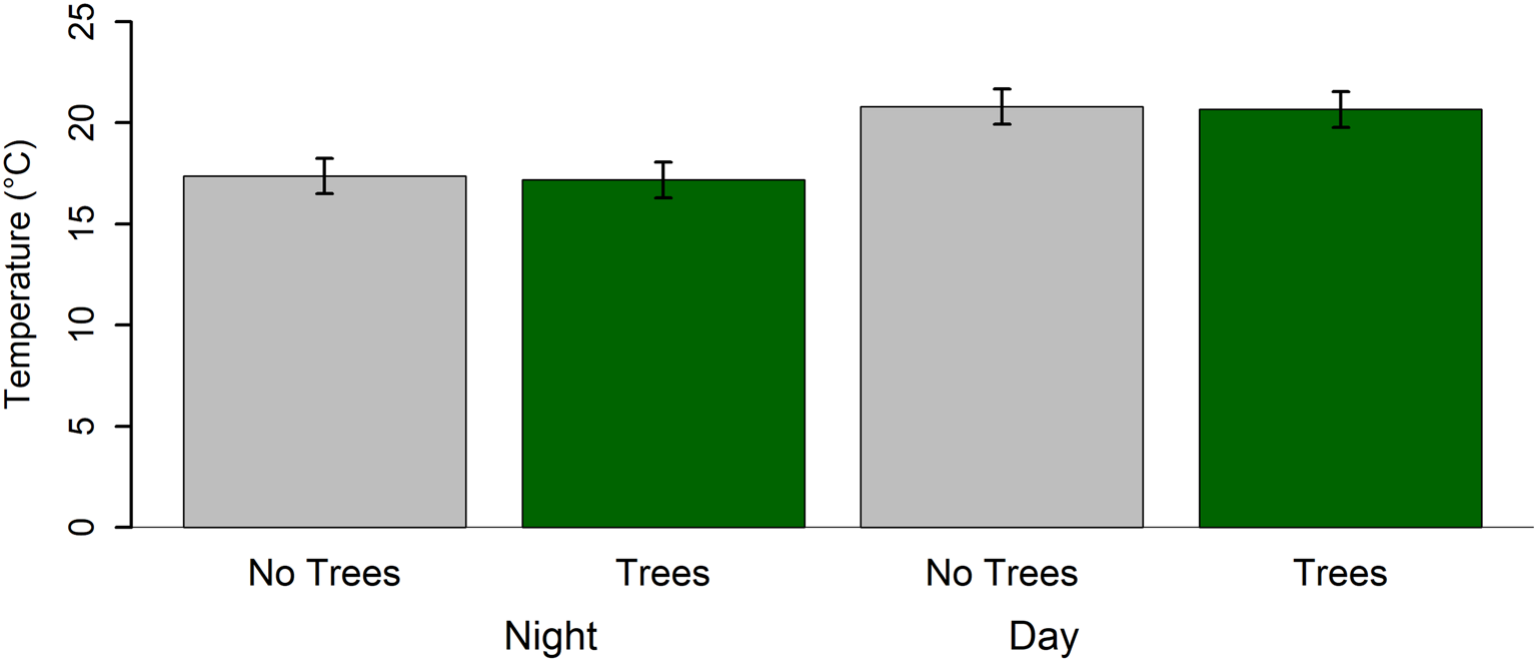
Cooling effects from tree canopy were consistent across summer days and nights (i.e., the interaction between canopy effects and day vs. night was not significant, Table 1)**,** and daytime temperatures were 3.45 °C cooler, on average, than nighttime temperatures. Bar plot shows estimates from model of hourly temperatures recorded from June through August 2022 by the 46 temperature loggers analyzed (see model summary in Table 1). Error bars represent 95% confidence intervals.

Within the focal South Tacoma neighborhood, we found that summer air temperatures were 3.45 °C warmer during the day than at night, on average, June through August 2022. Temperatures recorded at the same time varied among locations by 2.57 °C on average (range = 0.38–10.31 °C). This spatial among-location variation was similar during the day (mean = 2.58 °C, range = 0.38–10.31 °C) and night (mean = 2.57 °C, range = 0.38–8.93 °C, Fig. 2). We identified 24 unique tree genera located near our temperature loggers. Tree genera richness within 10 m of each logger ranged from 1 to 6; the most abundance genus was *Pseudotsuga* (Douglas-fir, Table S1).

We found that increased tree canopy cover was associated with lower hourly temperatures by 0.01 °C per % increase in field-measured canopy cover within 10 m (Table 1). Based on this modeled relationship, increasing from no tree cover to 100% tree cover at a given location would lead to a predicted drop in temperature of 1.0 °C at that location; increasing to 50% tree cover would lead to a drop of 0.50 °C. Comparing models with different field-measured metrics of tree abundance (presence, number of trees, basal area, canopy cover) provided similar explanatory power, based on conditional R^2^ (0.566 for all models, varying by less than one thousandth of a point, Table S2) and root mean squared error (which was 0.999 for all models, varying by less than one one-hundred thousandth of a point).

**Table 1:** Canopy cover effects on summer hourly temperature, based on a hierarchical model with predictors of field-measured canopy cover, day/night, elevation, an interaction between canopy cover and day/night, and intercept-only random effects of date and location (utility pole number).

Predictor	Air temperature (°C)		
	Estimate	95% CI	p
Intercept	**17.634**	**16.787 to 18.480**	** < 0.001**
Canopy cover (%)	**− 0.006**	**− 0.008 to − 0.003**	** < 0.001**
Day	**3.450**	**3.396 to 3.503**	** < 0.001**
Elevation	< 0.001	− 0.005 to 0.005	0.950
Canopy cover × day	0.001	− 0.001 to 0.003	0.605
Random effects			
σ^2^	12.10		
τ_00__Date_	12.72		
τ_00 Pole_No_	0.03		
ICC	0.51		
N_Date_	92		
N_Pole_No_	46		
Observations	88,124		
Marginal R^2^/conditional R^2^	0.108/0.566		

*p*-values < 0.05 are in bold.

**Figure 3:**
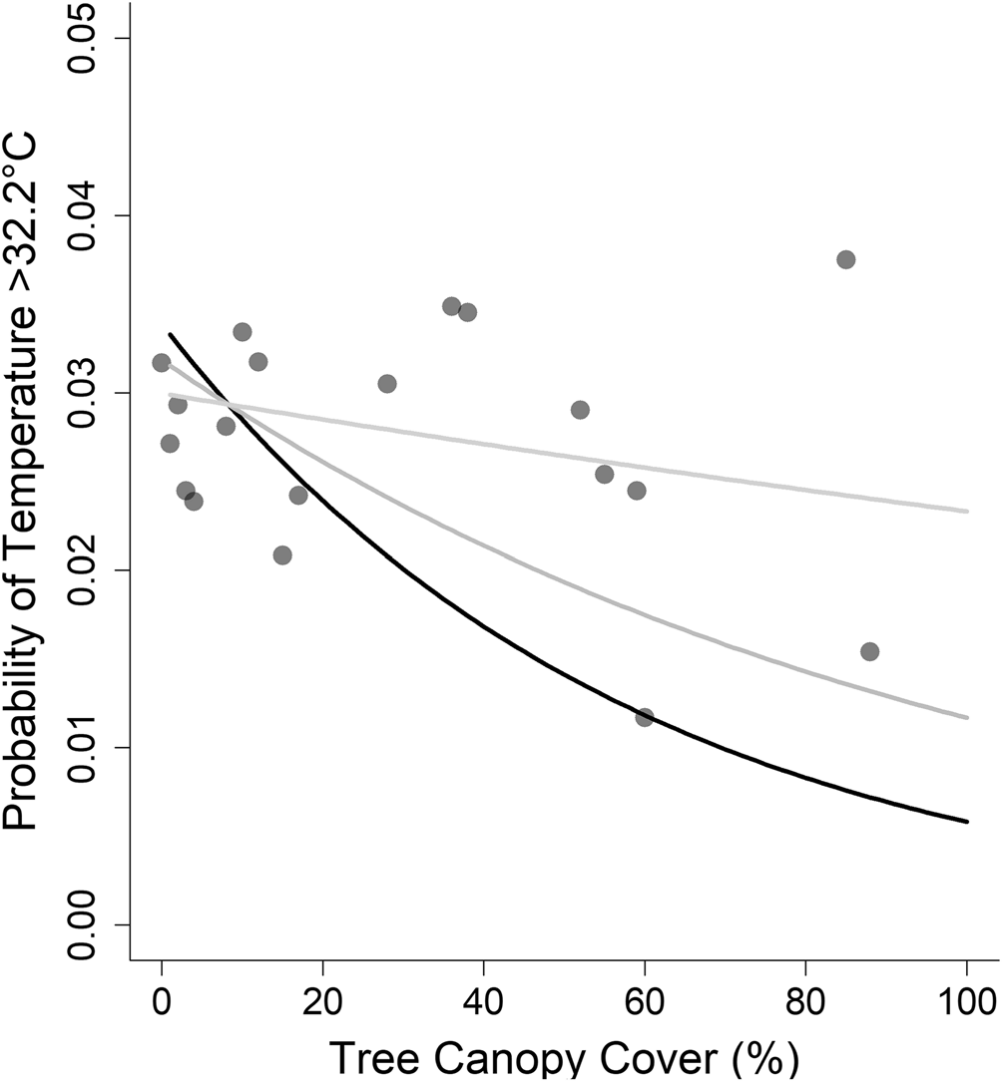
Probability of high heat events (> 32.2 °C) declined as canopy cover increased; the effect was weaker at higher elevations (i.e., the model included a significant interaction between canopy cover and elevation). To visualize this interactive effect, we show three lines, each representing a different elevation: the black line represents the estimated relationship between probability of high heat and canopy cover at 40 m above sea level; dark gray line is 60 m above sea level, and the light gray line is 80 m above sea level. Points represent the probability of high heat events at each location where we measured temperature (i.e., each utility pole). See Table 2 for model summary statistics, and Fig. S1 for a similar plot, using a high heat threshold of 26.7 °C.

Increasing tree canopy cover was associated with a reduction in the probability of high temperature events (Figs. 3 and S1, Table 2). This effect weakened slightly with rising elevation, which ranged from 35 to 110 m above sea level at locations where our temperature loggers were placed (mean = 84.9 m). The probability of temperatures exceeding the threshold value of 32.2 °C at elevations of 0 m, for example, was more than two times greater in locations with no tree canopy cover compared to those with 50% canopy cover and more than five times greater compared to those with 100% tree canopy cover (i.e., 0.03 versus and 0.01, 0.006, respectively, Fig. 3). At elevations of 60 m, this probability was 2.3 times greater in locations with no tree canopy cover than those with 100% tree canopy cover (i.e., 0.02 versus 0.04, Fig. 3). In summer 2022, the probability of daytime hourly air temperature measurements reaching or exceeding 32.2 °C was 0.029 (95% CI 0.027, 0.030). At least one of our loggers recorded maximum air temperature above this threshold on 11 days during summer 2022. If 2 °C warming is applied evenly to all locations, our model projects this probability would more than double, to 0.064 (95% CI 0.061, 0.066) or 23 days.

**Table 2:** Model summary for logistic regression model estimating probability of occurrence of high heat during summer days. Response was a Bernoulli variable representing whether or not temperature exceeded 26.7°C and 32.2 °C and predictors were field measured tree canopy cover, elevation, and their interaction.

Predictor	> 26.7 °C			> 32.2 °C		
	Odds ratio	95%CI	p	Odds ratio	95%CI	p
Intercept	**0.17**	**0.13–0.21**	** < 0.001**	**0.04**	**0.02–0.07**	** < 0.001**
Canopy cover	**0.99**	**0.98–1.00**	**0.013**	**0.97**	**0.95–0.99**	**0.001**
Elevation	1.00	1.00–1.00	0.251	1.00	0.99–1.00	0.314
Canopy cover × elevation	**1.00**	**1.00–1.00**	**0.029**	**1.00**	**1.00–1.00**	**0.002**
Observations	44,021			44,021		

*p*-values <0.05 are in bold

Temperatures in the study area were generally warmer than those recorded at nearby weather stations, and this difference was greater for minimum than maximum daily temperatures. Maximum temperatures were 0.73 °C warmer, on average (range = − 10.99 to 2.94 °C) and minimum temperatures were 1.05 °C warmer, on average (range = − 3.68 to 6.15 °C), in the study area compared to the weather station data. Cloud cover and relative humidity were not significantly associated with maximum temperature anomalies but canopy cover was an important predictor for minimum temperature anomalies (Table 3). A limitation of our study is that we did not quantify humidity alongside temperature at each utility pole; in addition, data collection in other seasons and years would be helpful in better understanding how conditions in the study area differ from weather stations.

**Table 3﻿:** Model summaries from hierarchical models estimating effects of tree canopy cover on maximum and minimum air temperature anomalies (°C) during summer 2022, using field-collected and remote-sensed assessments of canopy cover, with cloud cover data from nearby weather stations, and including an interaction term. Models also included intercept-only random effects of date and location (utility pole number).

Predictor	Field-based canopy cover (%)						Remote-sensed canopy cover (%)					
	Estimate		95%CI		p		Estimates		95%CI		p	
Maximum air temperature (°C)												
Intercept	**0.719**		**0.562 to 0.876**		** < 0.001**		**0.683**		**0.532 to 0.833**		** < 0.001**	
Canopy cover	**− 0.006**		**− 0.009 to − 0.003**		** < 0.001**		**− 0.010**		**− 0.015 to − 0.005**		** < 0.001**	
Cloud cover	0.002		− 0.001 to 0.004		0.13		0.002		− 0.000 to 0.005		0.093	
Canopy × cloud	< 0.001		− 0.000 to 0.000		0.333		< 0.001		− 0.000 to 0.000		0.818	
Random effects												
σ^2^	0.35						0.35					
τ_00 Date_	0.16						0.16					
τ_00 Pole_No_	0.06						0.06					
ICC	0.39						0.38					
N_Date_	92						92					
N_Pole_No_	50						50					
Observations	3506						3506					
Marginal R^2^/conditional R^2^	0.036/0.408						0.047/0.411					
Minimum air temperature (°C)												
Intercept	1.638		1.430 to 1.847		** < 0.001**		1.573		1.371 to 1.775		** < 0.001**	
Canopy cover	− 0.009		− 0.013 to − 0.004		** < 0.001**		− 0.012		− 0.020 to − 0.004		**0.002**	
Cloud cover	− 0.011		− 0.014 to − 0.007		** < 0.001**		− 0.01		− 0.013 to − 0.007		** < 0.001**	
Canopy × cloud	< 0.001		0.000 to 0.000		** < 0.001**		< 0.001		0.000 to 0.000		** < 0.001**	
Random effects												
σ^2^	0.17						0.17					
τ_00 Date_	0.27						0.27					
τ_00 Pole_No_	0.14						0.15					
ICC	0.7						0.7					
N_Date_	9						92					
N_Pole_No_	50						50					
Observations	3506						3506					
Marginal R^2^/conditional R^2^	0.177/0.756						0.170/0.755					

*p*-values < 0.05 are in bold.

**Figure 4:**
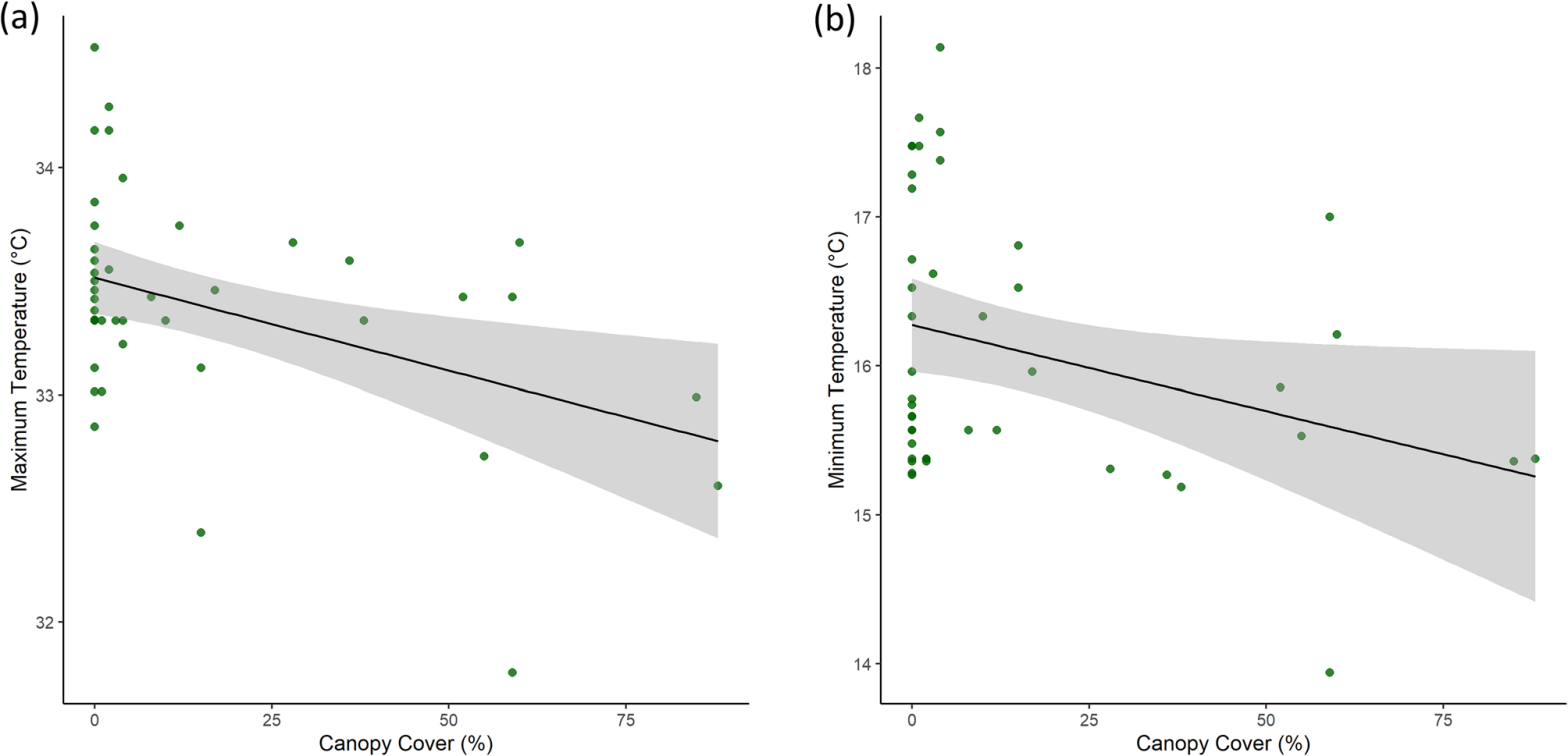
Tree canopy cover had linear negative effects on daily temperature maximum (**a**) and minimum (**b**) temperatures, shown here on one of the hottest days in our dataset, June 27, 2022. Effects of canopy on temperature were generally linear, though varied somewhat from one day to the next (Fig. S3).

Maximum and minimum temperature anomalies in the study area decreased linearly with increasing canopy cover (Fig. 4﻿). We found that minimum and maximum daily temperature anomalies were similarly affected by tree canopy cover: temperatures were reduced by 0.01 °C for minimum and maximum temperatures, per 1% increase in tree cover, based on remote-sensed estimates of canopy cover (Table 3). Thus, similar to the hourly analysis discussed above, we found that increasing from no tree cover to 100% tree cover would lead to predicted drops of 1.2 °C and 1.0 °C for minimum and maximum temperature, respectively. The strength of temperature-canopy cover relationships varied somewhat from day to day, but were generally linear, based on visual assessment (Fig. S3) and quantitative comparisons of daily relationships (i.e., linear relationships provided model fits with lower root mean squared error than nonlinear relationships on 68.4% of days in our study; linear models were also more likely to exhibit significant relationships [p < 0.05]).

**Figure 5:**
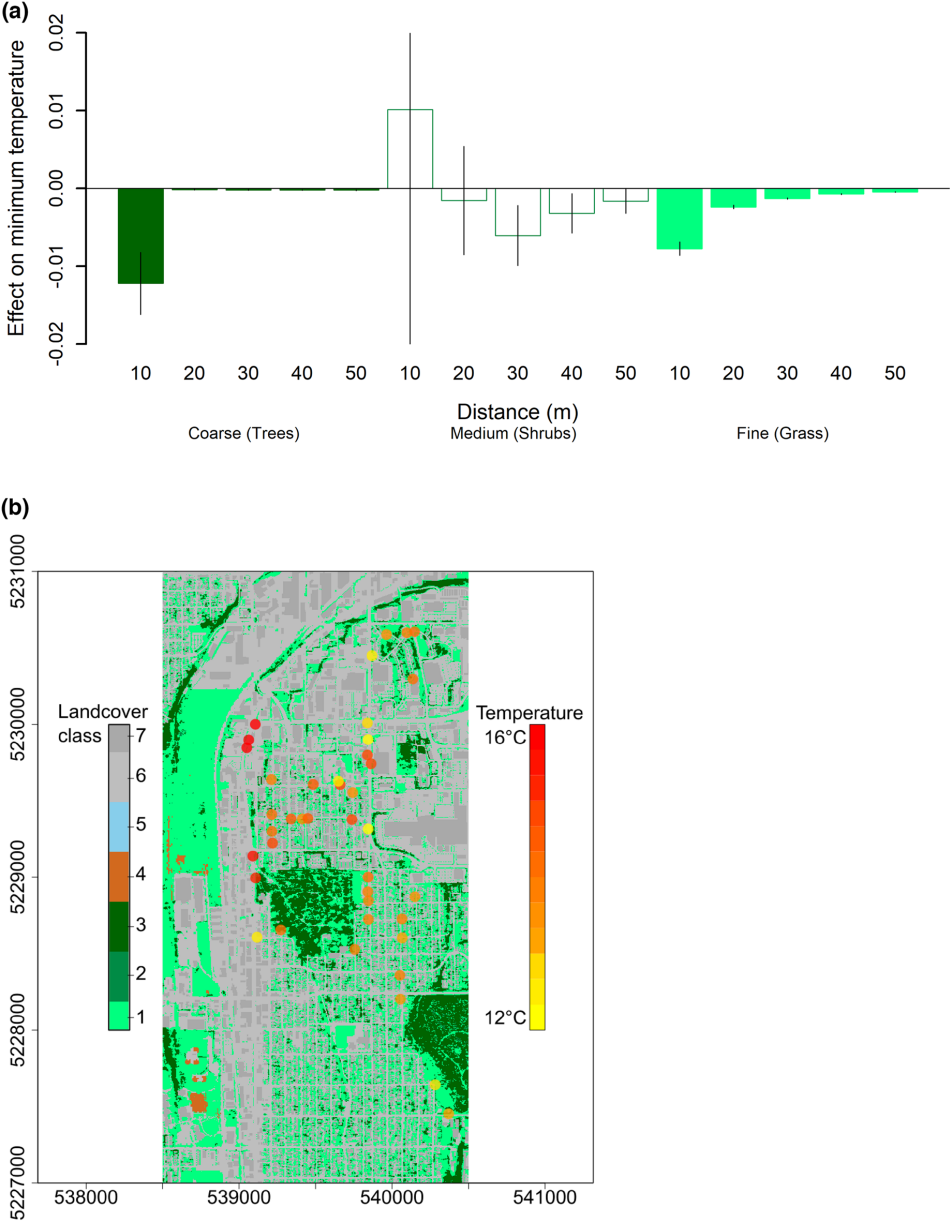
Effects on minimum and maximum daily temperature were strongest within 10 m and for trees (**a**), compared to further distances and other vegetation types (medium, fine) within different distances of temperature loggers. Land cover types in the study area (**b**), derived from remote-sensed data (available at 1 m^2^ resolution, https://www.stormwaterheatmap.org/), were compared to on-the-ground-measurements of tree cover taken at locations of temperature loggers (circles, color coded by mean minimum daily temperature). Vegetation classes are “Fine vegetation” such as grass (cover class 1), Medium vegetation” such as shrubs (cover class 2), and tree cover (“Coarse vegetation”, cover class 3), as well as dirt barren (class 4), water (class 5), impervious other (class 6), and impervious roofs (class 7). Effect sizes are from hierarchical linear models in Table S3; bars are filled if p < 0.10; error bars represent standard error. Map units are UTM Eastings and Northings.

Remote-sensed metrics of tree cover provided similar predictions of air temperature compared to field measured canopy cover. Some differences were apparent between the two metrics (Fig. S4), perhaps because the remote-sensed metrics we used were derived from 2018 data (5 years old), whereas field-measured data were current (collected in 2022 as part of this study), in addition to methodological differences in the two detection approaches. Nonetheless, the similarity in estimates of canopy cover effects on temperature (i.e., 95% uncertainty intervals overlap, Table 3) suggests that, when field data are not present, remote-sensed data can be used to estimate effects of tree cover on air temperature. Trees within 10 m had stronger effects on temperature than other types of vegetation in landcover dataset (i.e., medium or fine, Fig. ﻿5, Table S3). For both minimum and maximum temperature canopy cover quantified within 10 m were better predictors than canopy metrics at greater distances away (i.e., at distances of 20–50 m from the temperature logger location), and trees had stronger effects on temperature than other vegetation across all scales ranging from within 10–50 m of temperature logger locations (Fig. 5, Table S3).

## Discussion

We quantified variation in air temperature associated with tree canopy cover and show that the probability of crossing a temperature based human health threshold is two to five times greater in places with no trees versus full tree cover within 10 m. Moreover, we found the effect of tree canopy was linear in our focal neighborhood, so increasing tree cover by planting trees is likely to decrease local air temperature. Our research reveals that variation in tree cover within a neighborhood impacts the temperatures that people experience. It also highlights the need for increasing tree cover as climate change continues to exacerbate summer heat stress, especially in neighborhoods experiencing thermal inequities.

Many previous studies have focused on urban–rural or citywide comparisons; our work adds a finer resolution- within a neighborhood- to demonstrate that enhancing tree canopy can offer cooling benefits at very local scales. This work assesses field-measured air temperature, rather than modeled estimates, surface temperature data, or data collected from stationary weather stations. By measuring air temperature along sidewalks, we hoped to more accurately capture the heat experienced by people in their neighborhoods. Trees can also influence heat stress through their effects on humidity^29^, which we did not measure in this study. Though Pacific Northwest summers are typically low in humidity, high moisture conditions did play a role in the 2021 Pacific Northwest heatwave and may be increasingly important under climate change^30^.

The magnitude of variation in temperature we observed (2.57 °C) as well as differences in probability of high heat events in our focal neighborhood were similar to reported differences along urban vs rural comparisons^27,30^. For example, a study in Madison, Wisconsin, USA^30^ reported that urban areas experienced up to twice as many hours > 32.2 °C and temperatures up to 1.8 °C higher than rural areas, and a synthesis of 50 U.S. cities estimated urban–rural differences ranging from − 1.4 (urban cooling) to 1.5 °C, with a mean of 0.37 °C. The one study we found that occurred at a similar resolution as ours was also located in Madison, Wisconsin, and estimated that increasing canopy cover from 0 to 100% within a 10-m radius corresponded to a mean decrease of 0.7 °C in daytime air temperature^31^, slightly less than what we estimated in Tacoma (1.0 °C). Our work also adds to a growing body of research highlighting that trees specifically provide the strongest cooling effects compared with other vegetation types, e.g.,^31,32^. The large within neighborhood variation observed here and elsewhere, as well as the fact that the temperatures we recorded were generally warmer than those recorded at nearby weather stations, highlight that the urban heat experienced by people may be greater than what is commonly reported. This finding is particularly important for urban planners and managers to keep in mind while preparing urban environments for a warmer climate.

The linear, rather than nonlinear, relationship we find between local tree cover and air temperature suggests that there is not a clear threshold tree cover required to affect air temperature in our study area. Thus, every unit of added tree canopy cover can help to reduce local air temperature on hot days in South Tacoma and increasing canopy cover through tree planting is likely to lead to cooling in those locations. This differs from the Madison, Wisconsin study, which found that temperature decreased non-linearly with increasing canopy cover, with the greatest cooling occurring when canopy cover was greater than 40%^31^. At finer scales (i.e., within 10 m) within their study, however, relationships between canopy cover and temperature were more linear, as well. Temperatures within their study were greater than in ours (mean = 28.12 °C vs 19.16 °C in our study), with a narrower range (19.06–35.05 °C vs 3.26–46.98 °C in Tacoma). These differences may help explain our divergent findings, since variation in the magnitude or shape of relationships between tree cover and temperature may be due to the different urban contexts, species differences, or climatic differences, among other factors^33–35^.

Our research supports the important role of trees as infrastructure for urban climate change adaptation, given the relief they can provide from hot summer air temperatures. The cooling effects of tree canopy on temperature and their reduction on probability of high temperature events reported here (e.g., 0.01 °C per 1% increase in canopy cover, probability of exceeding 32.2 °C = 0.029 overall), may appear small. However, summer 2022 tied the previous record for number of days greater than 32.2 °C (90° F), previously set in 2015^36^. Even though the frequency of high temperature events is lower in our study area than in many other areas, these events can be extremely harmful when they occur. This is in part *because* of their relatively infrequent occurrence: insufficient heat acclimatization is a risk factor associated with heat-related illness morbidity^37^. Populations in cold climates are more sensitive to heat than those in warmer climates, and northern latitude cities, like Tacoma, have greater warm temperature-mortality risk than southern latitude cities^38^. Given the region’s historically mild summer temperatures, mechanical air conditioning is lacking in many Pacific Northwest households and residential landlord and tenant codes do not require provisioning of shading or air conditioning^39^. The June 2021 Pacific Northwest extreme heat event exemplified how dangerous these events can be for residents of this region, resulting in deaths of nearly 1000 people and heat-related illnesses harming thousands more^40,41^.

Such extreme heat events often hit hardest in low-income neighborhoods and neighborhoods of color^24,25^, the same neighborhoods where tree canopy tends to be lowest^19,42^. Our research was conducted in a neighborhood that exemplifies this low access to green infrastructure: tree canopy in the South Tacoma neighborhood where we conducted this research is 9%- less than half the city-wide average of 20%, only 1% of the land area is in parks versus 16% across the city, and 31% of people live below the poverty line (triple the county rate^43^). These disparities mean that the cooling benefits of trees are often not accessible to those most in need. Addressing these inequities is an urgent environmental justice issue, especially because extreme heat events are expected to become more frequent and intense over the coming decades^44^.

This study builds on the large body of research on tree canopy benefits in urban areas, adding new insight into the role of trees along sidewalks in affecting variation in temperature at fine scales, in a neighborhood where people live and work. Trees offer a viable nature-based solution to help address current and future urban heat problems. Maintaining and increasing tree canopy through tree protection, management, and planting can be accomplished through equitable, inclusive, and community-engaged approaches, though sufficient support is critical and urban tree programs are often under resourced^45–47^. We encourage planners and policy makers to support local planting efforts and community engagement around urban trees. Further, increasing tree canopy coverage through tree planting offers not only climate change adaptation, such as cooling neighborhoods and reducing energy needs, but also mitigation, through sequestration of carbon dioxide^48^. Thus, street trees and other urban trees can be seen as a valuable opportunity to mitigate urban heat, reduce risk of high heat exposure, and help mitigate climate change.

## Methods

### Data

Tacoma, Washington, is a city of 219,346 in the Pacific Northwest region of the United States^49^. It has a warm-summer Mediterranean climate (Köppen Csb), with the hottest temperatures occurring in July and August when average high temperatures are 25 °C. This research focused on the Tacoma Mall Regional Growth Center^43^ and surrounding neighborhoods, located in South Tacoma (Fig. 5). Conditions in this area are typical of many overburdened communities experiencing environmental injustice in the United States. It contains fewer green amenities than many other Tacoma neighborhoods: currently, the area has 9.1% tree canopy (compared to 20% citywide), with approximately 1% of the land area in parks (compared to 16% across the city); 70% of land cover is impervious (versus 52% citywide), including paved or hard surfaces such as streets, parking lots, and roofs^43^. Life expectancy for people in this area is 6 years shorter than the county average (visible at https://www.tpchd.org/healthy-people/health-equity/communities-of-focus/south-tacoma), and 31% of people live below the poverty line (triple the county rate^43^). These environmental injustices are particularly concerning because the area has been identified as a growth center and is zoned to become one of Puget Sound’s most dense urban centers^50^. This projected increase in human population in a nature-deprived area highlights the urgency behind understanding how urban tree canopy affects temperature.

In spring 2022, HOBO® pendant temperature sensors in solar radiation shields (https://www.onsetcomp.com/) were installed on 53 utility poles across the study area (Fig. 1). The sensors were installed at heights of 1.5–2 m above the ground surface on the sidewalk facing side of the poles, as the goal was to quantify temperatures that humans are likely to experience. Instantaneous measurements were recorded every hour. Vandalism, theft, and other damage to temperature loggers resulted in 46 utility poles where temperature data were available during summer 2022. We focus here on summer temperature (June, July, and August), as these are the hottest months in our study area and summer heat stress poses an increased occupational and public health risk with climate change. Although our study focuses on temperature, it is important to remember that other micro-climatic factors, including humidity, wind, and sun exposure, also affect heat stress^15,29,51^. Washington state law, based on current available evidence in the state, e.g.,^37,52^, triggers protective action (such as providing shade, or other sufficient means for cooling down) for construction and other outdoor workers at temperatures of 80° F (26.7 °C, with additional requirements (e.g., mandatory 10-min cool-down rest period every two hours) triggered at 90° F (32.2 °C, https://lni.wa.gov/rulemaking-activity/AO21-33/2133Adoption.pdf). We therefore used these values as thresholds for dangerously high temperatures, and calculated probability of temperatures being greater than these two thresholds.

In 2022, we recorded the identity (at the genus level) and size (diameter at breast height) of all trees within 10 m of each temperature sensor, and quantified tree canopy cover in the field using a densiometer. We were thus able to quantify tree abundance surrounding each temperature logger in three different ways based on field-collected data: number of trees, basal area, and canopy cover. We measured pre-existing street trees within the study area; no plant material was collected or manipulated, and the research complies with local, national, and international guidelines.

In addition to field collected data, we used temperature, cloud cover, and humidity data from nearby weather stations, available at https://www.visualcrossing.com/ and we quantified tree cover and finer scale vegetation cover (such as grass and shrub cover) using the land cover classes and 1 m^2^ resolution data available at https://www.stormwaterheatmap.org/. For temperature data, we calculated anomalies for each logger compared to weather station data for maximum and minimum daily temperature. For landcover data, we calculated the proportion of 1 m^2^ pixels that were tree cover (Cover Class 3, “Coarse vegetation”), as well as “Medium vegetation” such as shrubs (Cover Class 2) and “Fine vegetation” such as grass (“Cover class 1), within 10, 20, 30, 40, and 50 m radii of the utility pole locations (latitude, longitude) where temperature loggers were installed.

### Statistical analyses

We used hierarchical models to quantify effects of trees on temperature. This modeling framework allowed us to account for non-independence of sites and days (through “random﻿” effects) while testing for effects of tree cover and other variables (“fixed” effects) on temperature^53^.

To quantify effects that trees have on summer air temperature, using different field-measured metrics of tree abundance (presence, number of trees, basal area, and canopy cover), we fit models with Gaussian response variables of hourly air temperature, and predictors of tree presence/abundance, elevation, daytime versus nighttime, and the interaction between tree presence/abundance and day/night effects. We included intercept-only random effects of date and utility pole, as described by the following equation:

$${Air\, temperature}_{i}={y}_{i}={\mathrm{\alpha }}_{date\left[i\right]}+{\mathrm{\alpha }}_{pole\left[i\right]}+{{\beta }_{tree}x}_{tree\left[i\right]}+{{\beta }_{elev}x}_{elev\left[i\right]}+{{\beta }_{day}x}_{day\left[i\right]}+{{\beta }_{tree:day}{x}_{tree\left[i\right]}x}_{day\left[i\right]}+ {\varepsilon }_{i},$$

$${\varepsilon }_{i}\sim N\left(0,{\sigma }_{y}^{2}\right).$$

We compared the explanatory power of models fit with these different metrics of tree abundance using R^2^ , root mean squared error (RMSE), and Akaike’s Information Criterion (AIC).

To quantify how trees affect the probability of high temperatures occurring at local scales, we fit a generalized linear model with a Bernoulli response variable of hourly occurrence of high temperature (≥ 26.7 °C and 32.2 °C) during the day (6 am to 6 pm) and predictors of field measured tree canopy cover, elevation, and their interaction. This model can be written as:

$${\text{log}}(\frac{{p}_{i}}{1-{p}_{i}})={\mathrm{\alpha }}_{i}+{{\beta }_{tree}x}_{tree\left[i\right]}+{{\beta }_{elev}x}_{elev\left[i\right]}+{{\beta }_{tree:elev}{x}_{tree\left[i\right]}x}_{elev\left[i\right]}$$

where, *p* is the probability of temperatures reaching high temperature thresholds.

To understand the extent and scale at which broader landcover patterns affect air temperature in our focal neighborhood, we fit separate models with maximum and minimum daily temperatures as response variables. Predictors were vegetation cover, designated in the landcover dataset as coarse (i.e., trees), medium (shrubs), or fine (grass, herbs) at different distances from the temperature loggers (10 m, 20 m, 30 m, 40 m, 50 m). We fit separate models for each temperature variable (maximum or minimum) and predictors were each vegetation type-distance combination. For these models, we included intercept-only random effects of unique utility pole and used R^2^, RMSE, and AIC to compare model fit. We also visually and quantitatively compared linear and nonlinear models.

$${Air\, temperature \left(Max or Min\right)}_{i}={y}_{i}={\mathrm{\alpha }}_{date\left[i\right]}+{\mathrm{\alpha }}_{pole\left[i\right]}+{{\beta }_{tree}x}_{tree\left[i\right]}+{{\beta }_{cloud}x}_{cloud\left[i\right]}++{{\beta }_{tree:cloud}{x}_{tree\left[i\right]}x}_{cloud[i]}+ {\varepsilon }_{i}$$

$${\varepsilon }_{i}\sim N\left(0,{\sigma }_{y}^{2}\right).$$

All analyses were conducted in R Version 4.2.2^54^, and we used the lme4 package^55^ to fit hierarchical models.

### Supplementary Information

Supplementary Information.

## Data Availability

Code and data are available at KNB (https://knb.ecoinformatics.org/﻿)^56^.
