# Supplementary material for: Street trees provide an opportunity to mitigate urban heat and reduce risk of high heat exposure
Source: Sci Rep. 2024 Feb 13;14:3266. doi: 10.1038/s41598-024-51921-y (PMC10864265; doi:10.1038/s41598-024-51921-y)

Supplemental materials for "Street trees provide an opportunity to mitigate urban heat and reduce risk of high heat exposure"

Ailene K. Ettinger, Gregory N. Bratman, Michael Carey, Ryan Hebert, Olivia Hill, Hannah Kett, Phillip Levin, Maia Murphy-Williams, Lowell Wyse

Table S1. Tree genera in our study area, identified within 10m of each of the utility poles where temperature loggers are located. Genera are listed from most to least abundant, as quantified by total basal area (in m^2), summed across all surveyed areas. Frequency is the number of survey locations (utility poles) at which the species was present.

Genus	Basal Area (m^2)	Frequency
Pseudotsuga	19.262	4
Quercus	12.130	5
Acer	11.465	7
Prunus	3.443	10
Chamaecyparis	1.706	1
Unknown deciduous	0.674	4
Pinus	0.672	3
Robinia	0.585	1
Liquidumbar	0.558	3
Arbutus	0.390	2
Rhamnus	0.334	1
Crataegus	0.332	3
Syringa	0.314	10
Palm	0.196	1
Ulmus	0.157	1
Alnus	0.110	1
Rhodendron	0.084	1
Camillia	0.076	1
Fagus	0.045	1
Amelanchier	0.035	1
Thuja	0.020	1
Holodiscus	0.012	1
Unknown conifer	0.005	1

Table S2. Comparison of hierarchical model estimates of effects of trees (with different field-measured metrics of tree abundance) on hourly temperature**.** Models included predictors of trees (quantified by canopy cover, basal area, presence/absence, or number of trees within a 10 m radius of the temperature logger), daytime vs nighttime, elevation, and a tree-daytime interaction term, as well as intercept-only random effects of date and location (utility pole number). 95% confidence intervals (CI) are shown. AIC=Akaike's Information Criterion, RMSE=root mean squared error.

	**Canopy Cover (%)**			**Basal Area (m^2)**			**Presence**			**Number**		
Predictors	*Estimate*	*2.5% CI*	*97.5% CI*	*Estimate*	*2.5% CI*	*97.5% CI*	*Estimate*	*2.5% CI*	*97.5% CI*	*Estimate*	*2.5% CI*	*97.5% CI*
Intercept	17.6336	16.7895	18.4780	17.5445	16.6716	18.4175	17.3666	16.5002	18.2320	17.4123	16.5386	18.2855
Trees	-0.0058	-0.0085	-0.0031	-0.0257	-0.0728	0.0213	-0.1886	-0.3403	-0.0367	-0.0308	-0.0593	-0.0023
Day	3.4499	3.3962	3.5035	3.4916	3.4431	3.5400	3.4233	3.3458	3.5009	3.4131	3.3584	3.4678
Elevation	-0.0002	-0.0051	0.0047	0.0000	-0.0055	0.0056	0.0034	-0.0022	0.0090	0.0021	-0.0036	0.0077
Trees*Day	0.0005	-0.0014	0.0025	-0.0637	-0.0925	-0.0349	0.0521	-0.0441	0.1484	0.0257	0.0084	0.0431
**Random Effects**												
$\tau_{00\ Date}$	12.7220			12.7274			12.7263			12.7256		
$\tau_{00\ Pole\_No}$	0.0338			0.0452			0.0465			0.0505		
Observations	88124			88124			88124			88124		
Marginal R^2 / Conditional R^2	0.108 / 0.566			0.108 / 0.566			0.107 / 0.566			0.107 / 0.566		
AIC	470531.0			470511.0			470525.3			470527.7		
RMSE	0.9993			0.9992			0.9992			0.9992		

Table S3. Trees have a greater effect on temperature than other vegetation types and when nearby, based on effect sizes of vegetation from hierarchical model estimates of effects of coarse vegetation (trees), medium vegetation, and fine vegetation (quantified from remote-sensed data layers of land cover) on minimum temperature. The percentage of 1m^2 pixels containing each vegetation type was summed within circles with radii ranging from 10-50m around the locations of our temperature loggers. The largest significant effect sizes for each vegetation type, across the different radii examined, are shaded in gray; note that the effect of medium vegetation was not significant in any model we tested. Model estimates, their standard error (SE), and chi-square and *p*-values from Type III Wald χ^2 tests are shown. Significant model terms (p<0.05) are in bold font.

	Distance = 10m				Distance = 20m				Distance = 30m				Distance = 40m				Distance = 50m			
	Estimate	SE	χ^2	*p*	Estimate	SE	χ^2	*p*	Estimate	SE	χ^2	*p*	Estimate	SE	χ^2	*p*	Estimate	SE	χ^2	*p*
Intercept	**15.201**	**0.383**	**1571.31**	**<0.001**	**15.202**	**0.384**	**1570.161**	**<0.001**	**15.229**	**0.384**	**1574.382**	**<0.001**	**15.234**	**0.384**	**1574.649**	**<0.001**	**15.248**	**0.384**	**1574.704**	**<0.001**
Coarse vegetation	**-0.012**	**0.004**	**9.392**	**0.002**	**<0.001**	**<0.001**	**7.691**	**0.006**	**<0.001**	**<0.001**	**10.943**	**0.001**	**<0.001**	**<0.001**	**11.169**	**0.001**	**<0.001**	**<0.001**	**11.298**	**0.001**
Cloud cover	**-0.024**	**0.007**	**11.842**	**0.001**	**-0.024**	**0.007**	**11.854**	**0.001**	**-0.025**	**0.007**	**12.136**	**<0.001**	**-0.025**	**0.007**	**12.188**	**<0.001**	**-0.025**	**0.007**	**12.258**	**<0.001**
Coarse Veg*cloud cover	**<0.001**	**<.001**	**65.373**	**<.0.001**	**<0.001**	**<0.001**	**53.37**	**<0.001**	**<0.001**	**<0.001**	**75.561**	**<0.001**	**<0.001**	**<0.001**	**78.718**	**<0.001**	**<0.001**	**<0.001**	**70.181**	**<0.001**
Intercept	**15.042**	**0.386**	**1517.55**	**<0.001**	**15.046**	**0.386**	**1517.423**	**<0.001**	**15.075**	**0.386**	**1523.573**	**<0.001**	**15.069**	**0.386**	**1521.778**	**<0.001**	**15.062**	**0.386**	**1521.002**	**<0.001**
Medium vegetation	0.010	0.208	0.002	0.961	-0.002	0.007	0.049	0.824	-0.006	0.004	2.463	0.117	-0.003	0.003	1.593	0.207	-0.002	0.002	1.101	0.294
Cloud cover	**-0.023**	**0.007**	**10.551**	**0.001**	**-0.023**	**0.007**	**10.59**	**0.001**	**-0.023**	**0.007**	**10.933**	**0.001**	**-0.023**	**0.007**	**10.887**	**0.001**	**-0.023**	**0.007**	**10.821**	**0.001**
Med.Veg*cloud cover	-0.001	0.001	1.913	0.167	<0.001	<0.001	0.091	0.763	**<0.001**	**<0.001**	**22.796**	**<0.001**	**<0.001**	**<0.001**	**16.068**	**<0.001**	**<0.001**	**<0.001**	**12.357**	**<0.001**
Intercept	**15.767**	**0.392**	**1619.935**	**<0.001**	**15.813**	**0.39**	**1641.477**	**<0.001**	**16.051**	**0.389**	**1706.058**	**<0.001**	**16.059**	**0.39**	**1698.025**	**<0.001**	**16.015**	**0.392**	**1672.212**	**<0.001**
Fine vegetation	**-0.008**	**0.001**	**78.407**	**<0.001**	**-0.002**	**<0.001**	**108.709**	**<0.001**	**-0.001**	**<0.001**	**224.233**	**<0.001**	**-0.001**	**<0.001**	**197.794**	**<0.001**	**<0.001**	**<0.001**	**146.284**	**<0.001**
Cloud cover	**-0.030**	**0.007**	**17.994**	**<0.001**	**-0.03**	**0.007**	**17.875**	**<0.001**	**-0.032**	**0.007**	**20.759**	**<0.001**	**-0.032**	**0.007**	**20.67**	**<0.001**	**-0.032**	**0.007**	**20.01**	**<0.001**
Fine Veg*cloud cover	**<0.001**	**<0.001**	**318.087**	**<0.001**	**<0.001**	**<0.001**	**328.406**	**<0.001**	**<0.001**	**<0.001**	**484.913**	**<0.001**	**<0.001**	**<0.001**	**433.328**	**<0.001**	**<0.001**	**<0.001**	**360.417**	**<0.001**

Figure S1. Probability of high heat events (at least 26.7°C) declined as canopy cover increased; the effect was weaker at higher elevations (i.e., the model included a significant interaction between canopy cover and elevation). To visualize this interactive effect, we show three lines, each representing a different elevation: The black line represents the estimated relationship between probability of high heat and canopy cover at 40m above sea level; the dark gray line is 60 m above sea level, and the light gray line is 80m above sea level. Points represent the probability of high heat events at each location where we measured temperature (i.e., each utility pole). See Table 2 for model summary statistics and Figure 3 in the main text for a similar plot, using a high heat threshold of 32.2°C.

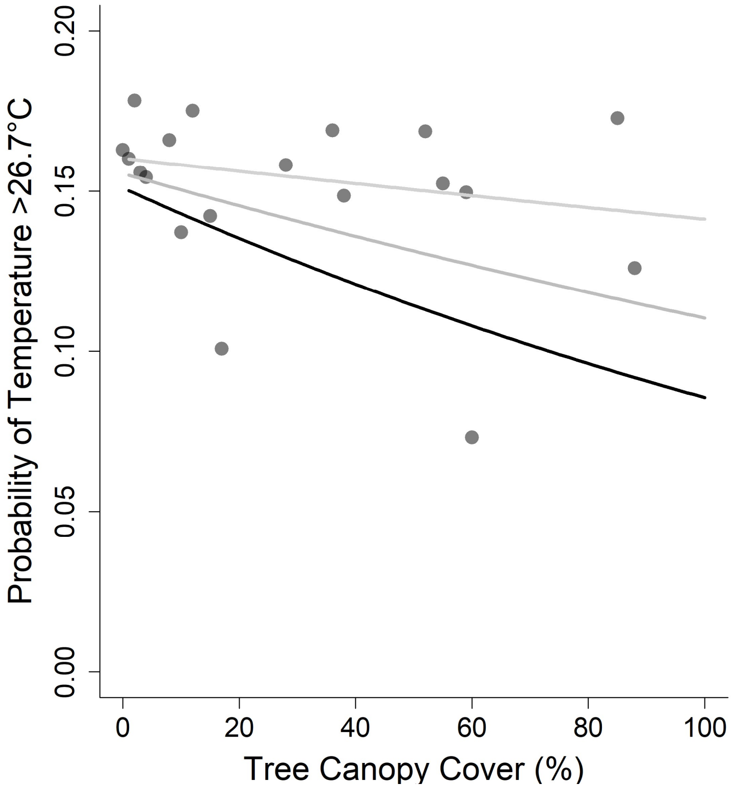

Figure S2. Effects of canopy cover on maximum (a, c) and minimum (b, d) temperature anomalies in our study area across June-August 2022. Top panels show field-based measurements of canopy cover; bottom panels show canopy cover estimates based on remote-sensed data layers.

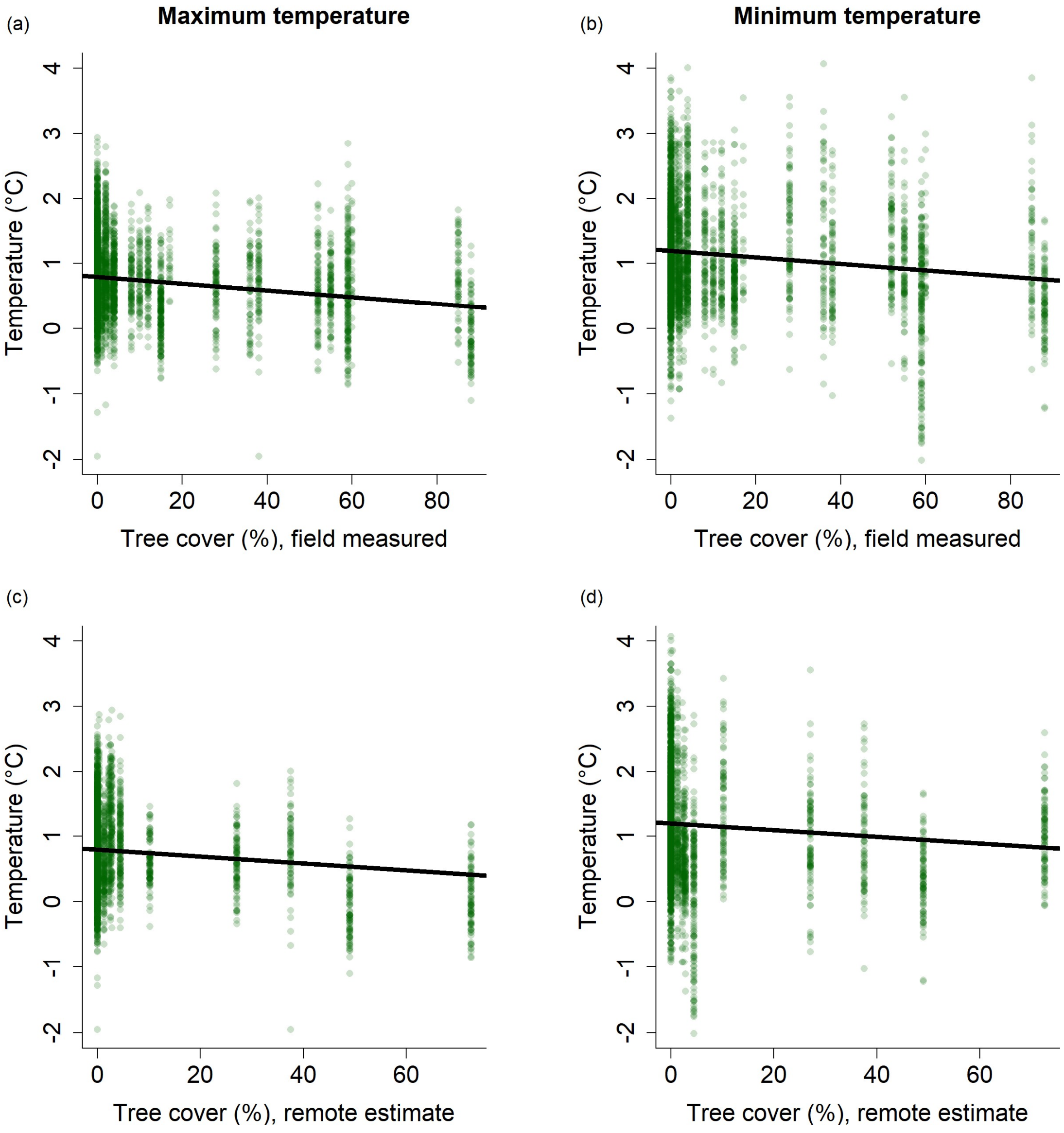

Figure S3. Relationships between tree canopy cover and air temperature were generally linear, and consistent across field and remote sensed canopy estimates. Plots of daily relationships between tree canopy cover and June, July, and August maximum (Tmax, left column) and minimum (Tmin, right column) daily air temperature show field-collected data (a, b, e, f, I, j) , and remote-sensed derived estimates of canopy cover (c, d, g, h, k, l). We quantitatively compared best-fit lines from linear models and nonlinear generalized additive models using a flexible spline approach and visually assessed relationships. Linear relationships provided model fits with lower root mean squared error than nonlinear relationships on 68.4% of days in our study and were more likely to exhibit significant relationships ($p<0.05$). Because patterns generally followed a linear relationship, we present and interpret linear hierarchical models in the main text.

(a) June Tmax, Field-based canopy cover

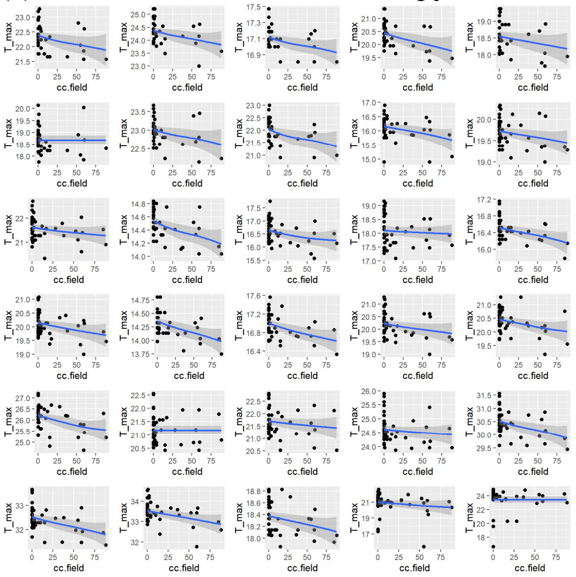

(b) June Tmin, Field-based canopy cover

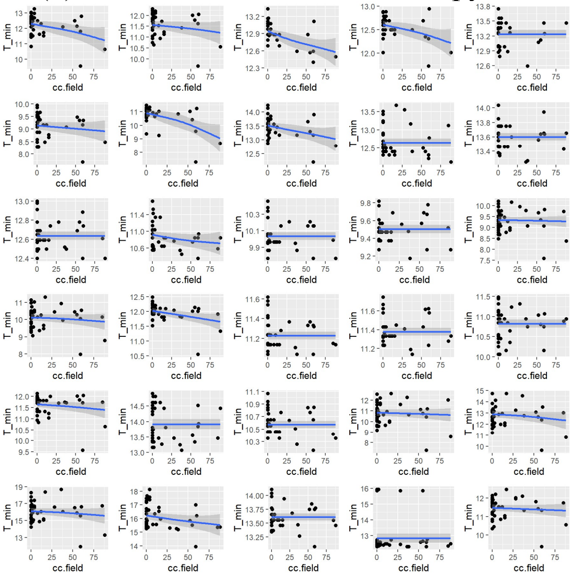

(c) June Tmax, Remote canopy cover

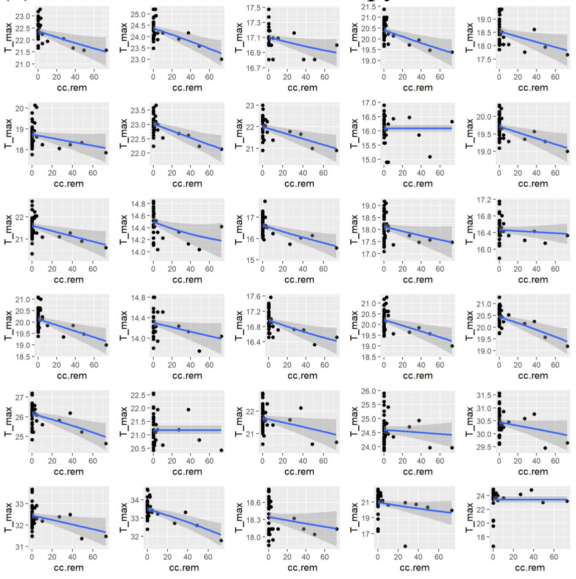

(d) June Tmin, Remote canopy cover

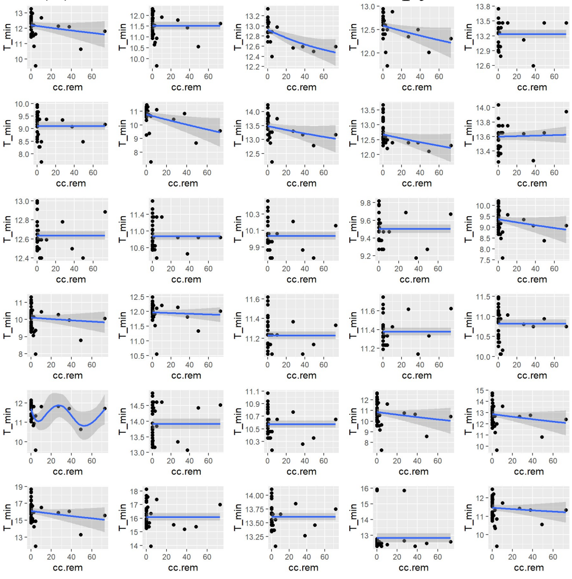

(e) July Tmax, Field-based canopy cover

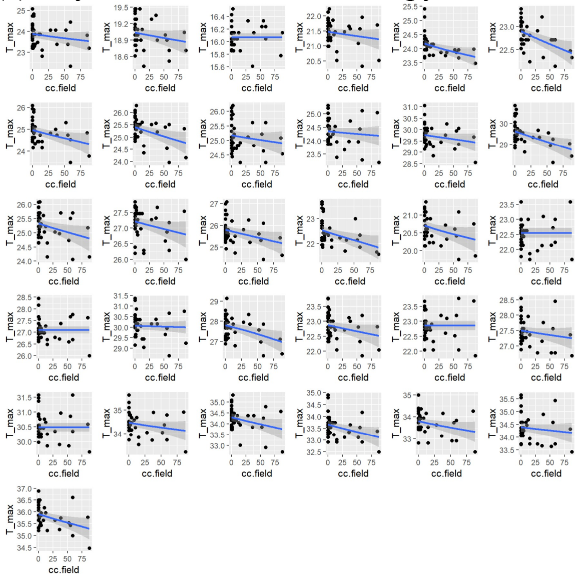

(f) July Tmin, Field-based canopy cover

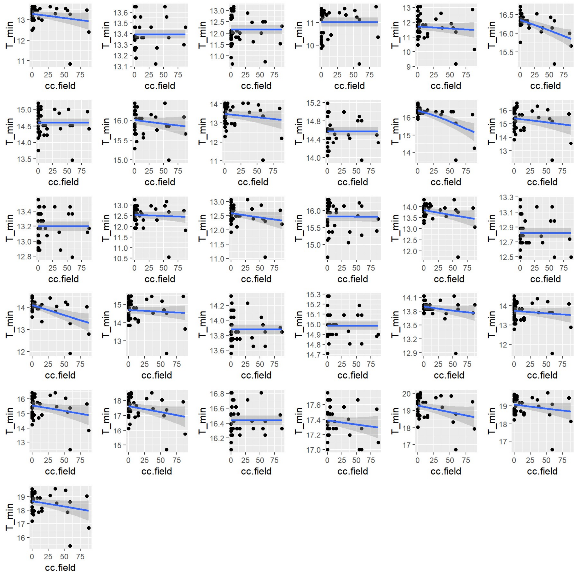

(g) July Tmax, Remote canopy cover

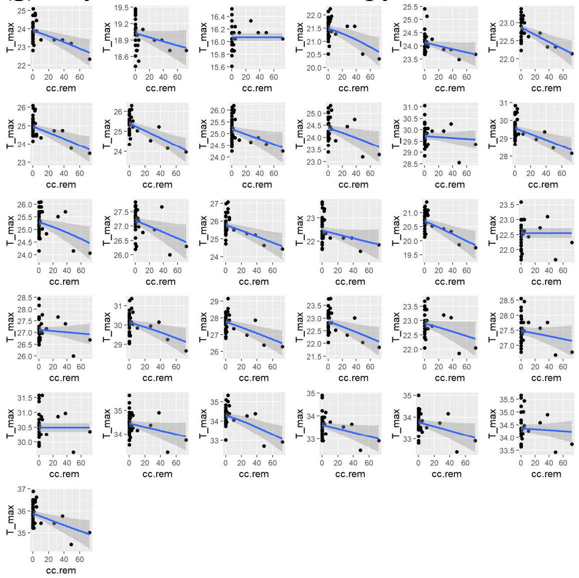

(h) July Tmin, Remote canopy cover

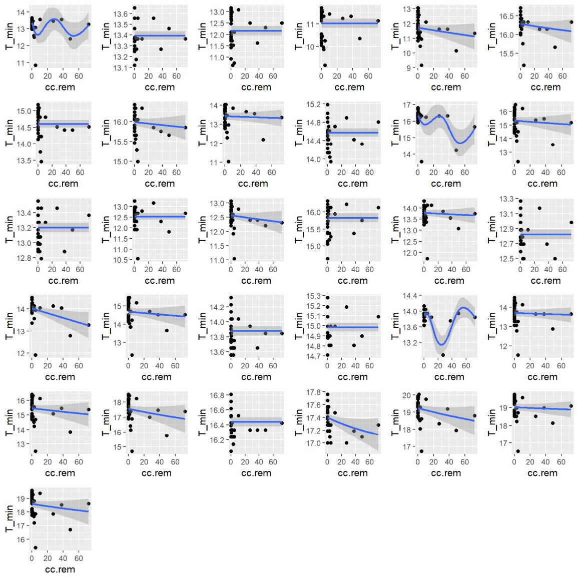

(i) August Tmax, Field-based canopy cover

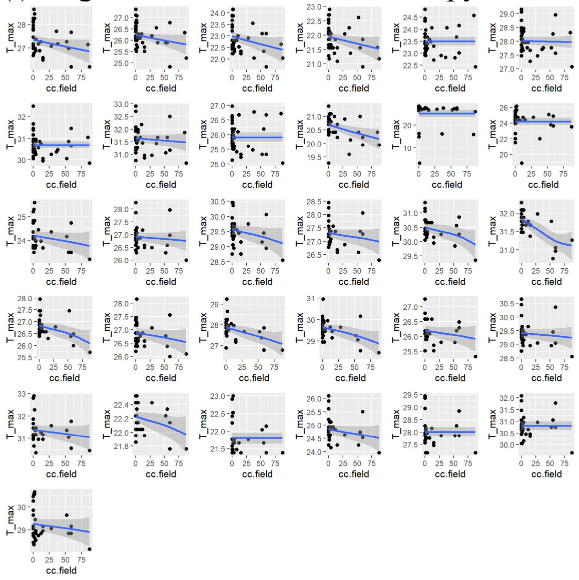

(j) August Tmin, Field-based canopy cover

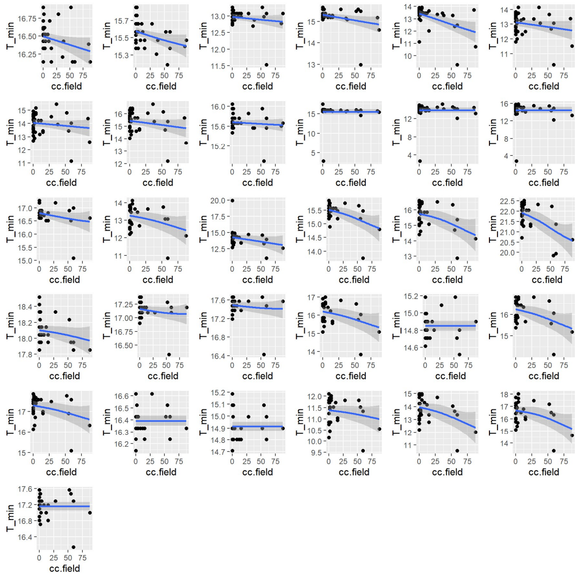

(k) August Tmax, Remote canopy cover

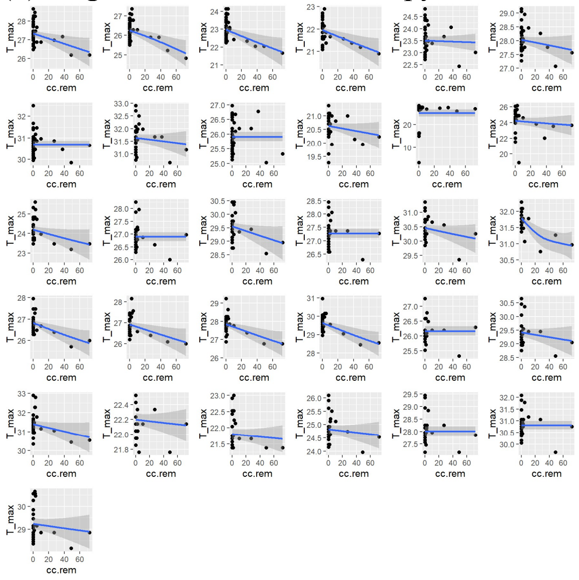

(l) August Tmin, Remote canopy cover

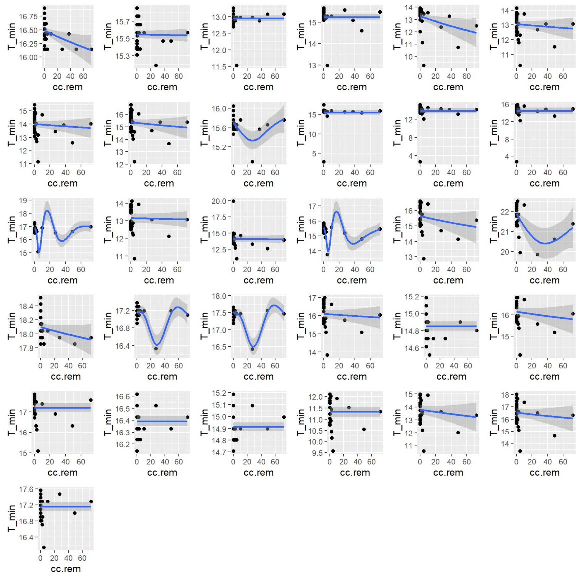

Figure S4. Field measured tree canopy cover (measured in 2022) is correlated with estimates of canopy cover derived from 2018 remote-sensed data, but there are differences across the two metrics. Solid line, r^2, and p-value represent the fitted linear model; dashed line represents the one-to-one line. Many remote-sensed estimates are lower than field-measured estimates, perhaps due to differences in the year of data collected (2018 for remote-sensed images versus 2022 for field collection), as well as the different methods (e.g., field measurements may more precisely quantify canopy from individual trees around the temperature loggers).

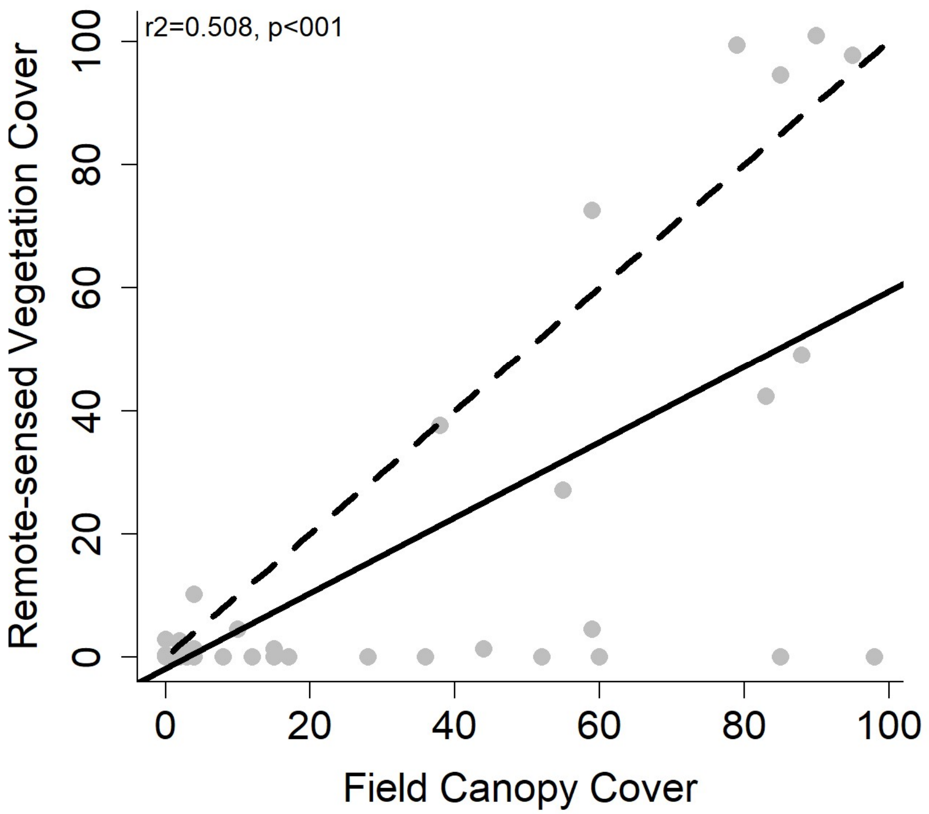

Supplement: Supplementary file 1 — Supplementary Information. [file 41598_2024_51921_MOESM1_ESM.pdf]
